# Speciation of Hexavalent Chromium in Aqueous Solutions Using a Magnetic Silica-Coated Amino-Modified Glycidyl Methacrylate Polymer Nanocomposite

**DOI:** 10.3390/ma16062233

**Published:** 2023-03-10

**Authors:** Ljiljana Suručić, Goran Janjić, Bojana Marković, Tamara Tadić, Zorica Vuković, Aleksandra Nastasović, Antonije Onjia

**Affiliations:** 1 Faculty of Medicine, University of Banja Luka, Save Mrkalja 14, 78000 Banja Luka, Bosnia and Herzegovina; 2 Institute of Chemistry, Technology and Metallurgy, University of Belgrade, Njegoševa 12, 11000 Belgrade, Serbia; 3 Faculty of Technology and Metallurgy, University of Belgrade, Karnegijeva 4, 11000 Belgrade, Serbia

**Keywords:** adsorption, amino-functionalized, glycidyl methacrylate, nanocomposite, quantum chemical modeling, silanization

## Abstract

A new magnetic amino-functionalized polymeric sorbent based on glycidyl methacrylate was synthesized and used in the separation of chromium Cr(VI) oxyanions sorption from aqueous solutions in a static batch system. The kinetic and isothermal parameters of the sorption process were determined. The experimental data were best fitted by a pseudo-second-order model with *R*^2^ = 0.994 and *χ*^2^ = 0.004. The sorption process of Cr(VI) removal by amino-functionalized sorbent was controlled by both intraparticle diffusion and liquid film diffusion. The equilibrium results showed that the sorption process is best described by the Freundlich model, followed closely by the Sips isotherm model, with a maximum sorption capacity of 64 mg/g. Quantum chemical modeling revealed that the sorption sites on the sorbent surface are fragments with diethylenetriamine and aminopropyl silane groups that coated the magnetic nanoparticles. The calculations showed that Cr(VI) oxyanions (Cr_2_O_7_^2−^, CrO_4_^2−^ and HCrO_4_^−^) bind to both sorption sites, with diethylenetriamine centers slightly favored. The X-ray photoelectron spectroscopy (XPS) spectra demonstrate that the chromium bound to the sorbent in the form of Cr(III), indicating that the Cr(VI) can be converted on the surface of the sorbent to a less harmful form Cr(III) due to the sorbent’s chemical composition.

## 1. Introduction

The predominant valence states of chromium in the environment are trivalent Cr(III) and hexavalent Cr(VI). Hexavalent chromium form has a variety of adverse effects on human health, including skin rash, weakened immune system, decreased pulmonary function, gastrointestinal and neurological issues, cytotoxicity, genotoxicity, etc., as opposed to trivalent chromium, which is biologically vital for the human body (25–35 µg/day) and influences sugar and lipid metabolism [1,2]. The main sources of Cr(VI) are leather and textile manufacturing, as well as the paint and pigment industry. According to the U.S. Environmental Protection Agency (EPA), the maximum permitted total chromium concentration in drinking water is 0.1 mg/dm^3^, while the World Health Organization (WHO) and Europe [Council Directive 98/83/EC on the quality of water intended for human consumption] limited allowable concentration at 0.05 mg/dm^3^ [3]. In recent decades, chromium contamination of water was a global issue, particularly in regions with intense mining, industrialization, and ineffective waste management, such as South Africa, Pakistan, China, and India. Recent research revealed an increase in Cr(VI) ions in India’s water systems where the mining industry developed [4,5], as well as an alarming 1.38-fold increase in China’s average chromium concentration compared to the background [6]. The fact that the yearly global production of chromium is over 10 million tons is concerning [7], considering its high solubility, mobility, and potential for bioaccumulation in human and animal tissues as well as plant life in terrestrial and aquatic ecosystems. Thus, Cr(VI), a prevalent heavy metal pollutant with significant adverse effects on human health, has been the subject of extensive research, as has its removal techniques from contaminated water [8,9]. A detailed preview of the current state of the art can be found in the literature [10,11]. Ion exchange methods, precipitation, solvent extraction, membrane separation, chemical and biological reduction, adsorption, and biosorption are some strategies that can eliminate the toxicity of this pollutant in the environment [12]. The main advantages of adsorption are high efficiency, speed, simplicity, and safety, as well as economic cost-effectiveness [13]. On the other hand, modern materials science faces a challenge in developing novel materials whose use could potentially improve existing treatment methods [14,15].

The nanosized magnetic heavy metal sorbents based on inorganic compounds or polymers attracted increasing attention in recent years [16,17,18]. Disadvantages of magnetic nanoparticles such as self-aggregation and oxidation in the air can be overcome by incorporation of non-leachable magnetite nanoparticles in the copolymer matrix in situ and surface coating with silanization agents such as alkoxysilanes and reagents with non-hydrolyzable amino groups [19,20,21]. Among the various organotrialkoxysilane molecules for the modification of silica surfaces, (3-aminopropyl)trimethoxysilane (APTMS) and (3-aminopropyl)triethoxysilane (APTES) were widely explored by many researchers [20,21,22,23]. The primary advantage of magnetic nanocomposites is that they can be easily removed using a simple magnet after sorption [16]. The excellent magnetic properties, as well as simple synthesis and functionalization, adaptable and controllable particle size, porosity and surface chemistry, chemical and thermal stability, biocompatibility, and permanent well-developed porous structure even in the dry state are particularly alluring [24,25]. Multifunctional, highly cross-linked macroporous glycidyl methacrylate-based non-magnetic and magnetic copolymers were successfully applied for decades in the sorption of heavy and precious metals, organic compounds, radionuclides, etc. [26,27,28].

In this study, a macroporous magnetic poly (glycidyl methacrylate-*co*-ethylene glycol dimethacrylate) (PGME) was prepared by in situ suspension copolymerization in the presence of Fe_3_O_4_ nanoparticles coated with APTMS as a silanization agent (Fe_3_O_4_@APTMS/PGME) and functionalized with diethylenetriamine, referred to as Fe_3_O_4_@APTMS/PGME-deta. Amino-functionalized nanocomposite was fully characterized in terms of their morphological, structural, and magnetic properties using scanning electron microscopy with energy-dispersive X-ray spectroscopy (SEM-EDX), transmission electron microscopy (TEM), Fourier transform infrared spectroscopy (FTIR), X-ray photoelectron spectroscopy (XPS), X-ray diffractometry (XRD), and SQUID magnetometry.

The main goal of this work is to explore the sorption properties of Fe_3_O_4_@APTMS/PGME-deta sorbent for removing Cr(VI) from aqueous solutions at unadjusted pH. Based on equilibrium, kinetic, and thermodynamic data, the mechanism of the Cr(VI) oxyanion sorption process on the sorbent Fe_3_O_4_@APTMS/PGME-deta was determined by monitoring the change in sorption capacity under the influence of various system parameters. The kinetic data were analyzed with pseudo-first-order (PFO), pseudo-second-order (PSO), Elovich, Avrami, and fractional power equations, while equilibrium data were analyzed using the Langmuir, Freundlich, Temkin, Toth, and Sips isotherm. The data were fitted by using a nonlinear regression method. Previously, we used FTIR and XPS to explain the oxyanions sorption mechanism onto amino-functionalized polymeric sorbents [25,29,30]. It was shown that oxyanions sorption was the consequence of electrostatic interactions and partial coordination. For a deeper understanding of the sorption mechanism and the Cr(VI) interactions with Fe_3_O_4_@APTMS/PGME-deta, in this study, intraparticle diffusion (IPD), Bangham, and liquid film diffusivity (LFD) models, as well as SEM-EDX, FTIR and XPS analysis before and after Cr(VI) sorption, were performed. Using statistical and quantum-chemical methods based on our previous studies, the binding energy of chromium oxyanions to active sites on the surface of the sorbent was calculated.

## 2. Materials and Methods

### 2.1. Materials

All chemicals used for magnetic sorbent synthesis were analytical grade products and were used as received. Glycidyl methacrylate (GMA), ethylene glycol dimethacrylate (EGDMA), diethylenetriamine (deta), 2,2′-Azobis(2-methylpropionitrile) (AIBN), cyclohexanol, and 1-tetradecanol were obtained from Merck (Darmstadt, Germany). Magnetite (nanopowder with particle size 50 nm, ≥98% trace metals basis) and APTMS were obtained from Sigma-Aldrich (Taufkirchen, Germany). Poly(N-vinyl pyrrolidone) (PVP, Kollidone 90) was purchased from BASF (Ludwigshafen, Germany). Toluene, methanol, and ethanol were obtained from Zorka Pharma (Šabac, Serbia). Potassium dichromate (K_2_Cr_2_O_7_, p.a. purity) was obtained from Merck (Darmstadt, Germany). Deionized water was used for the preparation of the solution.

### 2.2. Preparation of Magnetic Sorbent

#### 2.2.1. Silanization of Fe_3_O_4_ Nanoparticles by APTMS

Magnetite nanoparticles (5 g) were dispersed in a toluene/methanol solution (100 cm^3^ 50/50 by weight) and sonicated for 30 min. After that, 50 cm^3^ of solvent was evaporated, and 50 cm^3^ of methanol was added. This operation was repeated twice to remove excess water. Then, APTMS (5 cm^3^) was added to the suspension and stirred for 3 h at 323 K, then cooled down to room temperature, followed by decantation and thorough rinsing with ethanol. Finally, the obtained coated magnetic nanoparticles were dried in a vacuum at 333 K for 3 h and named Fe_3_O_4_@APTMS.

#### 2.2.2. Synthesis of Amino-Functionalized Magnetic Nanocomposite

Magnetic nanocomposite (Fe_3_O_4_@APTMS/PGME) was synthesized by suspension copolymerization of 29.2 g of GMA and 19.5 g of EGDMA and AIBN as an initiator (0.5 g) in the presence of an inert component (51.0 g of cyclohexanol and 12.8 g of tetradecanol) and 4.55 g of Fe_3_O_4_@APTMS. The monomer phase was sonicated at 300/600 W in an ultrasonic water bath Sonic 12 GT (Sonic, Niš, Serbia) and transferred in the aqueous phase that consists of 225.0 g of deionized water and 2.25 g of PVP. Copolymerization was carried out under an N_2_ atmosphere at 348 K for 2 h, then at 353 K for another 2 h with a stirring rate of 250 rpm. Synthesized copolymer particles were repeatedly rinsed with distilled water and ethanol, kept in ethanol for 12 h, and dried overnight in a vacuum oven at 323 K. The obtained Fe_3_O_4_@APTMS/PGME was classified with 0.15, 0.30, and 0.63 mm sieves.

A sample of magnetic nanocomposite (particles with diameters in the range of 0.15–0.30 mm) was additionally functionalized with diethylenetriamine, as described previously [31]. The resulting amino-functionalized magnetic nanocomposite was labeled as Fe_3_O_4_@APTMS/PGME-deta.

### 2.3. Characterization of Magnetic Sorbent

SEM-EDX analysis of Fe_3_O_4_@APTMS/PGME-deta was performed by scanning electron microscope using a JEOL JSM-6610LV instrument (JEOL Ltd., Tokyo, Japan). In order to make them conductive, samples were coated with a thin gold layer (the gold thickness was 15 nm) in a high-vacuum evaporator prior to scanning. TEM analysis was performed on the JEM-1400 Plus Electron microscope (JEOL USA, Inc., Peabody, MA, USA), with a voltage of 120 kV and LaB6 filament. FTIR spectra were recorded in ATR mode using a Nicolet 380 spectrometer (Thermo Scientific, Waltham, MA, USA) over the range of 400–4000 cm^−1^ with a resolution of 2 cm^−1^. XPS analysis was carried out using a SPECS, PHOIBOS100 spectrometer (SPECS Surface Nano Analysis GmbH, Berlin, Germany) and dual anode Al/Ag monochromatic source. The survey XPS spectra were taken using a monochromatic Al *Kα* line with an energy step of 0.5 eV in FAT 40 mode, while high-resolution spectra were taken with an energy step of 0.1 eV in FAT 20 mode. XRD patterns were recorded with an Ital Structure APD2000 X-ray diffractometer (G.N.R. S.r.l., Turin, Italy) in a Bragg–Brentano geometry using Cu *Kα* radiation (λ = 1.5418 Å) and step-scan mode (range: 10–70° 2*θ*, step-time: 1.0 s, step-width: 0.02°). The program Powder Cell (Federal Institute for Materials Research and Testing, Berlin, Germany) was used for approximate phase analysis. Field dependence of isothermal magnetization *M*(*H*) at *T* = 300 K was measured on a SQUID-based commercial magnetometer Quantum Design MPMS-XL-5 (Quantum Design, Inc., San Diego, CA, USA) in the applied DC fields up to 5 T.

The point of zero charge of Fe_3_O_4_@APTMS/PGME-deta was determined by the technique of evaluating the change of initial and equilibrium pH values [32]. For this purpose, the initial pH of 0.01 mol/dm^3^ NaCl solutions (20 cm^3^ in a series of erlenmeyer flasks) was adjusted between 2 and 11 using 0.1 mol/dm^3^ NaOH or 0.1 mol/dm^3^ HCl. Equilibrium pH was determined after adding 50 mg of sorbent and mixing for 24 h at room temperature [25]. The initial pH values were plotted against ΔpH to obtain the point where the initial pH equals the final pH (pH_PZC_).

### 2.4. Sorption Experiments

Amino-functionalized magnetic nanocomposite was evaluated as a Cr(VI) sorbent under static batch conditions at unadjusted pH (pH = 5.9) at room temperature (*T* = 298 K). Based on our previous studies, in order to determine the sorption rate, 0.2 g of Fe_3_O_4_@APTMS/PGME-deta was brought into contact with 20 cm^3^ (25 mg/dm^3^) of the Cr(VI) solution. For 60 min, Cr(VI) solutions (20 cm^3^) with concentrations ranging from 1 to 180 mg/dm^3^ were in contact with 0.2 g of Fe_3_O_4_@APTMS/PGME-deta to determine the sorption isotherm. For the sorption thermodynamics studies, additional experiments were performed at 298, 310, 329, and 343 K with an initial Cr(VI) ions concentration of 25 mg/dm^3^. At predetermined intervals, sample aliquots were extracted and analyzed using inductively coupled plasma optical emission spectrometry, ICP-OES (model iCAP 6500, Thermo Scientific, Waltham, MI, USA).

The amount of Cr(VI) sorbed per unit mass of magnetic sorbent, i.e., the sorption capacity, *Q* (mg/g), was determined using Equation (1):

(1) $$Q=\frac{(C_{i}-C)*V}{m}$$

where *C_i_* (mg/dm^3^) and *C* (mg/dm^3^) are the initial and final concentrations in the solution following sorption, respectively, *V* (dm^3^) is the volume of the solution, and *m* (g) is the mass of the magnetic sorbent.

### 2.5. Quantum Chemical Modeling

The optimization of possible dimers containing oxyanion and its sorption site (derivatives of deta, APTMS, and ethyl hydroxide) was performed in the Gaussian09 program (Gaussian, Inc., Wallingford, CT, USA) using B3LYP functional, 6–311 g ** basis set for non-metals and lanl2dz basis set for chromium. Optimized structures were used for the prediction of binding energies between the mentioned species on the same level of theory as well as optimization. The solvation energies were included and calculated by the implicit solvation model (SMD) (dielectric constant (*ε*) for water was 78.39). The binding energy values were calculated based on Equation (2):

(2) $$\Delta E_{V}=E_{O-A}-E_{O}-E_{A}$$

where: Δ*E_V_* is the energy of interaction within the dimer (or binding energy), $E_{O-A}$ is the energy of the optimized structure of dimer, *E_O_* is the energy of the optimized structure of oxyanion, and *E_A_* energy of the optimized structure of sorption site, which represents a model molecule for sorption center on sorbent surface. The basis set superposition error (BSSE) was included by using the standard (Boys–Bernardi) counterpoise procedure [33].

## 3. Results and Discussion

### 3.1. Characterization of Fe_3_O_4_@APTMS/PGME-Deta

The magnetic polymer sample functionalized with diethylenetriamine (Fe_3_O_4_@APTMS/PGME-deta) was porous with a pore diameter corresponding to half of the pore volume of 286 nm and a specific pore area value of 37 m^2^/g, as determined by mercury porosimetry [21]. The sample Fe_3_O_4_@APTMS/PGME-deta was also fully characterized using SEM, TEM, ATR-FTIR, XRD, and SQUID magnetometry.

**Figure 1:**
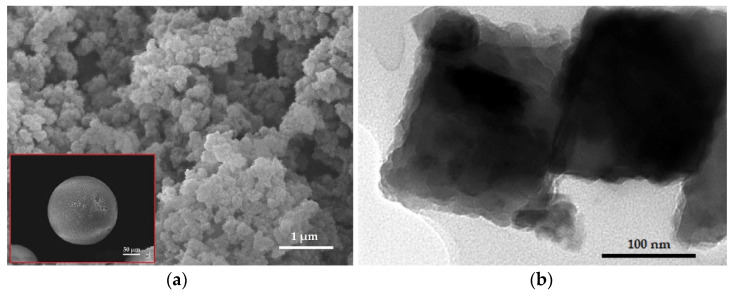
(**a**) SEM image of cross-section (WD 11 mm, scale bar 1 μm, magnification 20,000×) and (**b**) TEM image (horizontal field width 436.9 nm, scale bar 100 nm, magnification 120,000×) of Fe_3_O_4_@APTMS/PGME-deta. Inset: SEM image of Fe_3_O_4_@APTMS/PGME-deta bead (WD 11 mm, scale bar 50 μm, magnification 300×).

Figure 1a shows the SEM images of smooth spherical shape particles and cross-sections with the typical macroporous morphology formed by suspension copolymerization, consisting of the pores within microspheres, interstitial cavities between the microspheres, and pores between the agglomerates of the microspheres [34]. In addition, TEM image (Figure 1b) confirms the encapsulation of Fe_3_O_4_ nanoparticles (dark areas), which are dispersed in the gray copolymer matrix.

**Figure 2:**
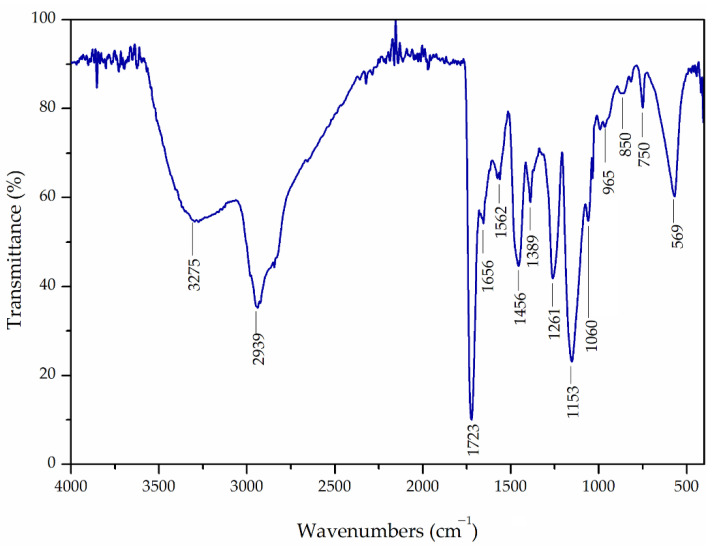
ATR-FTIR spectra of Fe_3_O_4_@APTMS/PGME-deta nanocomposite.

Grafting of the magnetite surface through silanization was confirmed by the ATR-FTIR spectrum (Figure 2). Namely, the band at 1060 cm^−1^ for Si-O-Si stretching, as well as stretching Si-O vibration at 965 cm^−1^ confirm the presence of silanized magnetite [20]. The strong peak originating from Fe-O vibrations at ~570 cm^−1^ confirms the successful incorporation of magnetite nanoparticles [35]. The characteristic bands for crosslinked methacrylate copolymer at ~2939 cm^−1^, ~1456 cm^−1^, and ~1389 cm^−1^ (methyl and methylene stretching and bending vibrations of C-H bond), 1723 cm^−1^ (C=O stretching vibrations), 1261 cm^−1^, and 1153 cm^−1^ (stretching C-O-C vibrations) also appeared in Fe_3_O_4_@APTMS/PGME-deta spectra [36,37]. The absorption bands for the epoxy ring vibrations at ~850 cm^−1^ and ~1260 cm^−1^ indicate incomplete functionalization of epoxy groups. Furthermore, the bands at 1562 cm^−1^ and 1656 cm^−1^ (stretching N-H vibrations of primary and secondary amine groups), 750 cm^−1^ (wagging N-H vibrations) as well the broad band at ~3700–3050 cm^−1^ (stretching N-H and O-H vibrations) confirmed the amino-functionalization [25,38].

**Figure 3:**
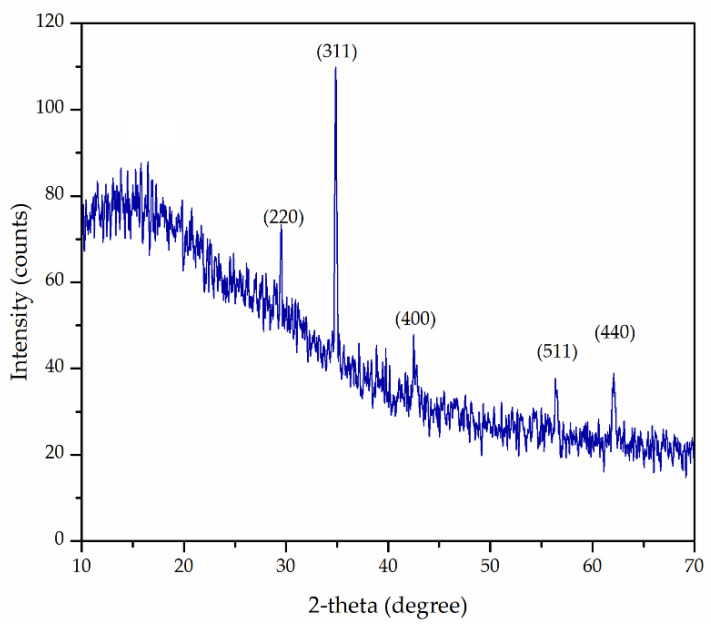
XRD pattern of Fe_3_O_4_@APTMS/PGME-deta.

The existence of magnetite phase Fe_3_O_4_ was confirmed in Fe_3_O_4_@APTMS/PGME-deta (Figure 3) by comparing the XRD data to the ICSD 65339 card. The unit cell parameter of Fe_3_O_4_ was *a* = 8.3661 Å. As one can see, the main characteristic diffraction peaks (2*θ* = 30.10, 35.42, 43.05, 56.94, and 62.15) corresponded to (220), (311), (400), (511), and (440) reflections of standard Fe_3_O_4_ crystal with spinal structure [39]. Additionally, the broad peak at 2*θ* = 10–30° indicated the existence of an amorphous polymer coating layer on the surface of iron oxide nanoparticles. A similar observation was reported for magnetic polymer-bentonite composite [19].

**Figure 4:**
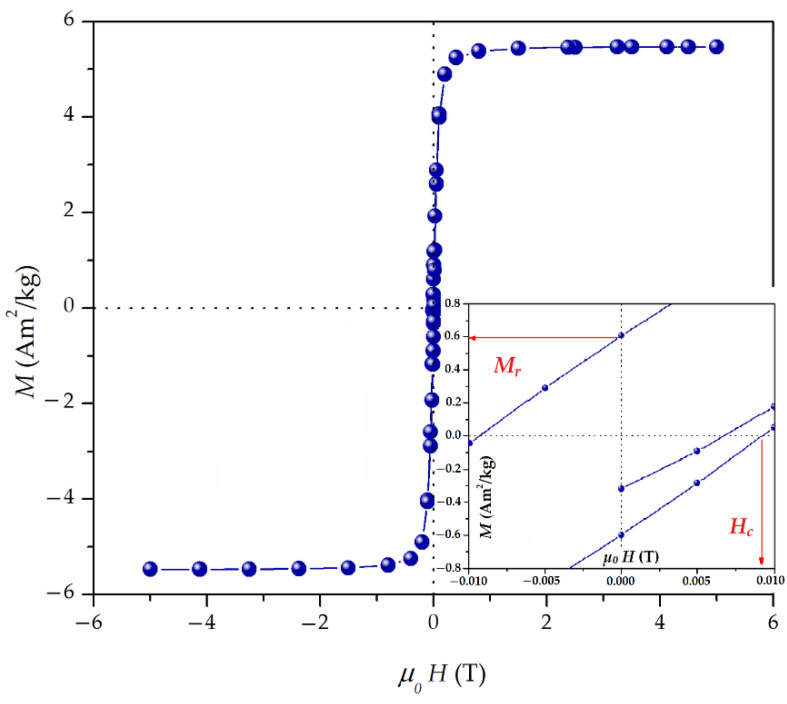
Magnetization curve at 300 K and ±5 T of Fe_3_O_4_@APTMS/PGME-deta. Inset: Magnified view of magnetization curve near the origin.

Figure 4 presents the magnetization curve of Fe_3_O_4_@APTMS/PGME-deta at 300 K. The values of remnant magnetization (*M_r_* = 0.6 Am^2^/kg) and coercivity (*H_c_* = 0.009 T) at 300 K (Figure 4 inset) suggested a superparamagnetic behavior of Fe_3_O_4_@APTMS/PGME-deta. The hysteresis loop *M*(*H*) curve showed a very fast increase in magnetization with the saturation magnetization value (*M_s_*) of 6.8 Am^2^/kg, indicating that the Fe_3_O_4_@APTMS/PGME-deta can be facilely separated from aqueous solutions by applying an external magnetic field and reuse.

**Figure 5:**
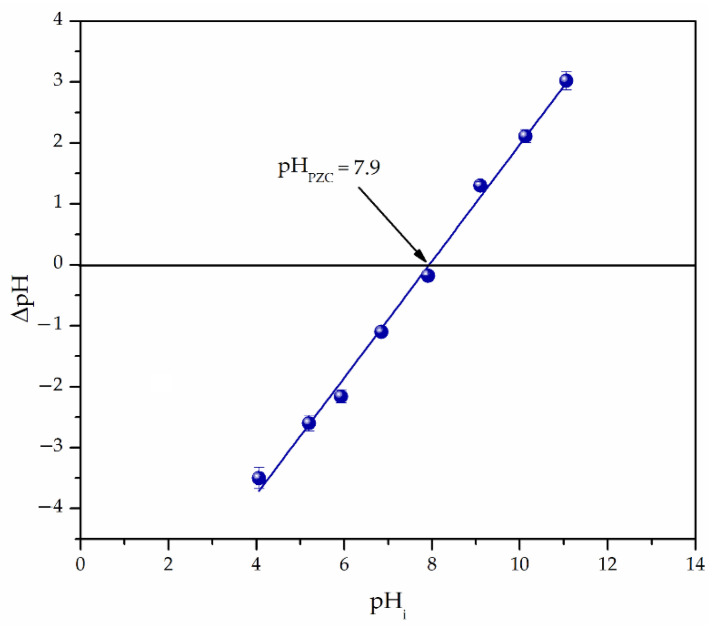
Point of zero charge (pHpzc) for Fe_3_O_4_@APTMS/PGME-deta. Standard errors are shown as vertical error bars.

Based on the pH_PZC_ value of 7.9 (Figure 5), the positively charged Fe_3_O_4_@APTMS/PGME-deta surface at pH < pH_PZC_ electrostatically attracted negatively charged Cr(VI) ions and, thus, enabled their efficient removal. On the other hand, due to electrostatic repulsion at pH > pH_PZC_, the negatively charged Fe_3_O_4_@APTMS/PGME-deta surface did not promote the Cr(VI) ions sorption.

### 3.2. Chromium Sorption onto Fe_3_O_4_@APTMS/PGME-Deta

Previously, Cr(VI) sorption was studied on non-magnetic amino-functionalized macroporous GMA-based copolymer from concentrated metal solutions (0.05 mol/dm^3^) and at low pH values (pH = 1.8) [40]. In this study, sorption capacity was tested on magnetic Fe_3_O_4_@APTMS/PGME-deta in diluted solutions with a concentration of 25 mg/dm^3^ (0.48 mmol/dm^3^). All experiments were performed at pH = 5.9 and 298 K, i.e., close to ambient conditions, in order to come as close as feasible to actual environmental concentrations.

The kinetic data were analyzed with theoretical models of PFO, PSO, Elovich, Avrami, and fractional power. The nonlinear regression method was used to fit the data to determine the best-fit kinetic model. For that purpose, two error functions, coefficient of determination (*R*^2^) and chi-square statistic test (*χ*^2^), were used for analysis. The used nonlinear fitting equations of the kinetic data are presented in Table S1.

**Figure 6:**
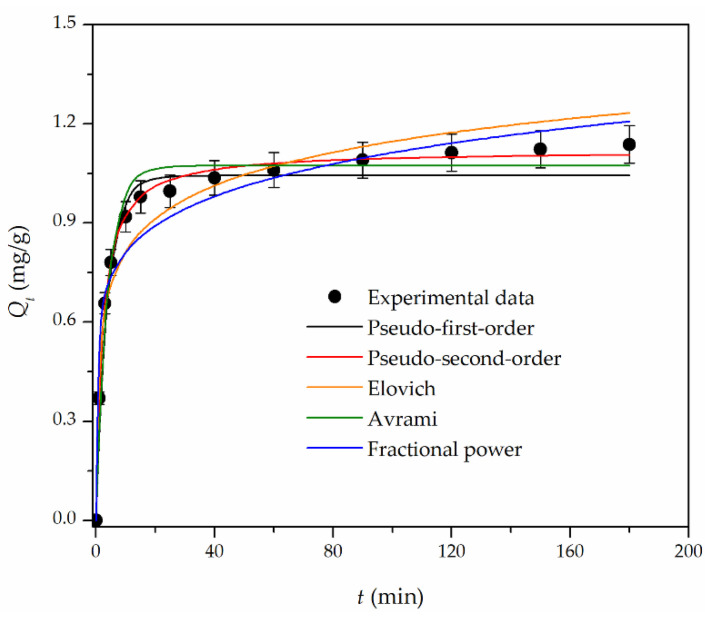
Nonlinear fitting curves for the kinetics sorption of chromium on Fe_3_O_4_@APTMS/PGME-deta sorbent. Error bars represent the standard error of three replicates.

As seen in Figure 6, the sorption at the initial stage was rapid (up to 10 min), and the process then gradually slowed down to 50 min when the uptake equilibrium and saturation was attained. The sorption half-time, *t*_1/2_ (the time required to reach 50% of the total sorption capacity), was approximately 2 min, and the sorbent saturation was achieved after 40 min. These results indicate that most Cr(VI) ions were sorbed at the binding sites of Fe_3_O_4_@APTMS/PGME-deta on and/or near the surface. The calculated kinetic parameters and the error calculation are summarized in Table 1.

Based on error function values (lowest *χ*^2^ and highest *R*^2^) calculated for different kinetic models, the fitting degree is as follows: PSO > PFO > Avrami > Elovich > fractional power kinetic models. Additionally, the experimental value of sorption capacity at equilibrium, *Q_e_^exp^*, (1.2 mg/g) is closer to the calculated value of equilibrium sorption capacity, *Q_e_^calc^*, for the PSO model (1.12 mg/g) than the value of the PFO (1.04 mg/g) and Avrami equation (1.07 mg/g). The experimental data are most consistent with the PSO kinetic model, indicating that the rate of sorption is dependent on the affinity of the sorbate to the sorbent, i.e., the oxyanion and properties of the magnetic nanocomposite. It also refers to chemisorption, which involves interactions that alter the electronic structure of the sorbent’s active site and the oxyanion [41].

For the study of the rate-controlling step of the chromium sorption process, four (IPD, Bangham, Boyd, and LFD) models were utilized. The used equations of the four diffusion models are presented in Table S1, while values of obtained relevant parameters are given in Table 2.

The IPD plot (Figure S1a) indicates three diffusion stages: boundary layer diffusion, intraparticle diffusion, and equilibrium stage, where IPD slows down [42]. However, all three straight lines of the fitting curve do not pass through the origin, indicating that intraparticle diffusion is not the only rate-controlling step of chromium sorption. The plots of Bangham and Boyd models (Figures S1b and S1c, respectively) confirmed this result [29,43]. LFD model assumes that the slowest stage of the sorption process is diffusion through the boundary layer of the sorptive solution [44]. Although the LFD plot (Figure S1d) deviated from the origin, the high *R*^2^ value (*R*^2^ = 0.943) suggested the definite influence of film diffusion on chromium sorption by Fe_3_O_4_@APTMS/PGME-deta sorbent.

**Figure 7:**
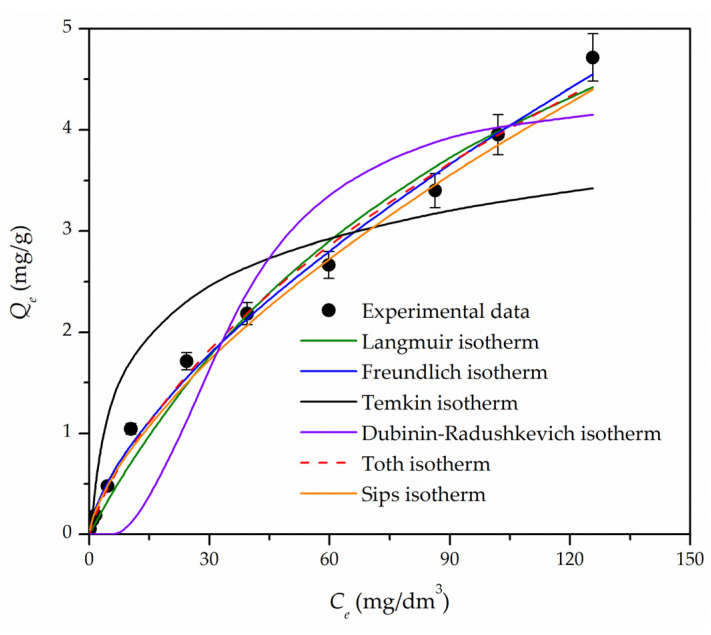
Nonlinear fitting curves for the adsorption isotherms of chromium onto Fe_3_O_4_@APTMS/PGME-deta sorbent. Error bars represent the standard error of three replicates.

The Langmuir, Freundlich, Tempkin, Dubinin–Radushkevich, Toth, and Sips isotherm models were used to describe the equilibrium data by employing nonlinear fitting forms (Table S2). The calculated values of parameters and the plot of six applied isotherm models are presented in Table 3 and Figure 7, respectively.

Considering *R*^2^ and *χ*^2^ values, the sorption isotherm models fitted the experimental data in the order of Freundlich > Sips > Toth > Langmuir > Dubinin–Radushkevich isotherm > Temkin isotherm. The Freundlich model describes heterogeneous systems and reversible sorption and is not restricted to the formation of monolayers. The value of *K_F_* is related to the degree of sorption, while the Freundlich constant (1/*n*) is a measure of the deviation of the sorption from linearity; the more heterogeneous the surface, the 1/*n* value is closer to zero [41]. The Freundlich constant obtained in this study was 0.65 (corresponding to an *n* value of 1.54), indicating favorable sorption. The value of the free energy of sorption calculated from the Dubinin–Radushkevich model was 59.5 kJ/mol, suggesting that the process of chromium removal onto Fe_3_O_4_@APTMS/PGME-deta was chemisorption governed by particle diffusion mechanism [45]. According to the Sips model, the maximum sorption capacity was 64.13 mg/g. Additionally, the Sips exponent (*m* = 0.68) was less than 1, indicating the predominance of chromium sorption on a heterogeneous surface.

The maximum Cr(VI) sorption capacity of Fe_3_O_4_@APTMS/PGME-deta obtained from the Sips isotherm model was compared with different magnetic sorbents having a wide range of sorption capacities (Table 4). The literature review focused on studies published in the last decade, and the choice was made according to the structural similarities of the sorbents with our sorbent in terms of functional groups and/or the incorporated coated and uncoated magnetite. Unlike the majority of sorbents listed in Table 4 that were tested in a highly acidic environment, Fe_3_O_4_@APTMS/PGME-deta was used at an unadjusted pH, closer to real conditions. In that manner, the waste production and secondary pollution were minimized, making Cr(VI) sorption process with Fe_3_O_4_@APTMS/PGME-deta low-cost, economical, and environmentally friendly. Bearing that in mind, it can be said that Fe_3_O_4_@APTMS/PGME-deta has a reasonable capacity for Cr(VI) sorption from aqueous solutions.

A thermodynamic investigation was conducted at four temperatures in the 298–343 K range. Table 5 shows the thermodynamic parameters of chromium sorption calculated from experimental data using Equations (3)–(5) [28]:

(3) $$K_{c}=\frac{C_{a}}{C_{e}}$$

(4) $$\Delta G^{0}=-RT\ln K_{C}$$

(5) $$\ln K_{C}=-\frac{\Delta G^{0}}{RT}=\frac{\Delta S^{0}}{R}-\frac{\Delta H^{0}}{RT}$$

where *C_e_* (mg/dm^3^) and *C_a_* (mg/dm^3^) are the concentration of chromium ions in solution at equilibrium and the amount of chromium ion sorbed onto Fe_3_O_4_@APTMS/PGME-deta at equilibrium, respectively, *R* (8.314 J/mol K) is the universal gas constant, *T* (K) the absolute temperature and Δ*G*^0^ (kJ/mol), Δ*S*^0^ (kJ/mol K), and Δ*H*^0^ (kJ/mol) are the change of standard Gibbs free energy, standard entropy, and standard enthalpy, respectively.

Negative Δ*G*^0^ values at all temperatures imply spontaneous sorption of chromium on the magnetic nanocomposite sorbent, but positive Δ*H*^0^ values suggest the process is endothermic. A positive value of Δ*S*^0^ indicates the sorbent’s affinity for chromium oxyanion and a higher probability of solid–liquid phase interactions leading to structural changes on both the magnetic nanocomposite and the oxyanion, which is characteristic of chemisorption.

### 3.3. Chromium Sorption Mechanism

**Figure 8:**
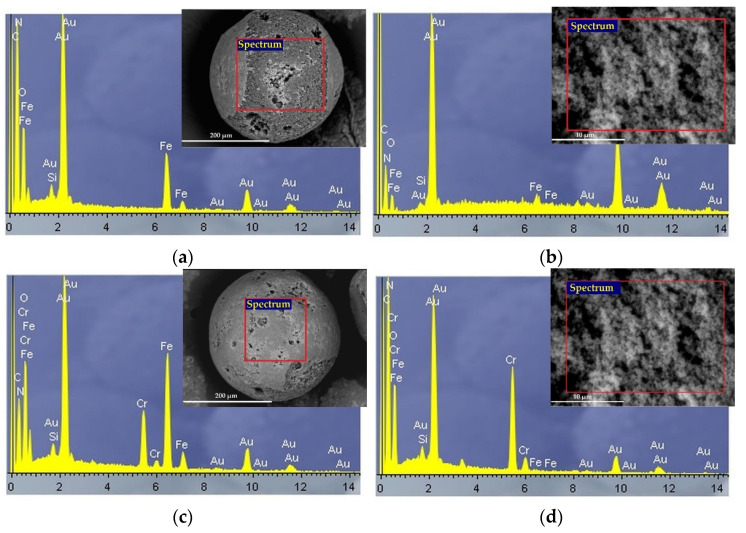
EDX spectra and SEM images for Fe_3_O_4_@APTMS/PGME-deta of (**a**) particle surface and (**b**) cross-section before sorption and (**c**) particle surface and (**d**) cross-section after chromium sorption.

**Figure 9:**
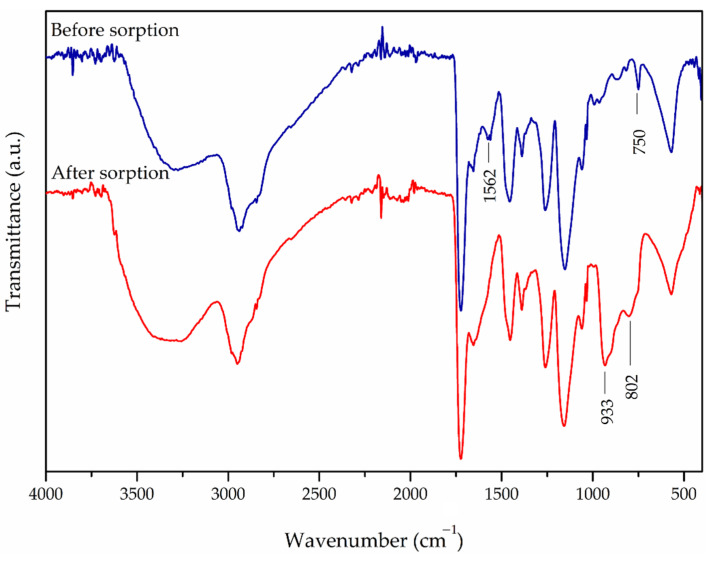
ATR-FTIR spectra of Fe_3_O_4_@APTMS/PGME-deta before and after sorption of chromium.

**Figure 10:**
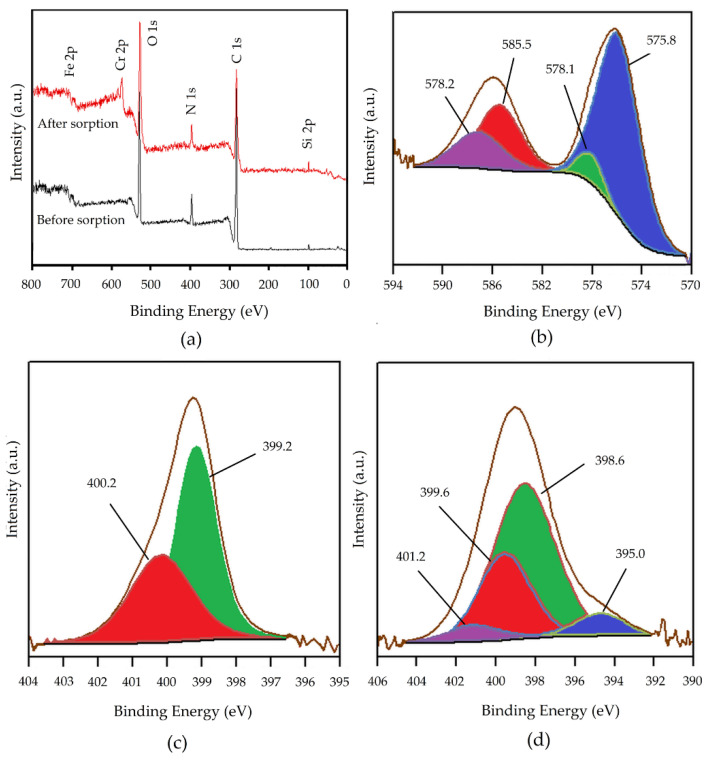
XPS spectra of Fe_3_O_4_@APTMS/PGME-deta: (**a**) total survey spectrum before and after sorption of chromium, (**b**) HRES Cr 2p, (**c**) HRES N 1s before and (**d**) HRES N 1s after sorption.

In order to understand the interaction mechanism between chromium oxyanions and Fe_3_O_4_@APTMS/PGME-deta, the SEM-EDX (Figure 8), FTIR (Figure 9) and XPS analyses (Figure 10) of the samples before and after sorption were performed.

The EDX spectrum confirmed the presence of all expected elements (C, O, N, Fe, Si, Cr). The N peaks showed an amino-functionalization, Fe peaks suggested the incorporation of magnetite, while the presence of the Si peak evidenced the silanization of magnetite particles. After sorption, the presence of a Cr peak was observed on the particle surface as well as on the cross-section, thus proving successful sorption. As shown in Figure 8d stronger Cr peak on the cross-section indicated that intraparticle diffusion has a great influence on sorption. Due to gold coating on Fe_3_O_4_@APTMS/PGME-deta, the EDX spectra show Au peak.

As can be observed from FTIR spectra, the intensity and position of some characteristic peaks of the magnetic nanocomposite sorbent after chromium sorption were changed. After chromium sorption, two new peaks at 933 cm^−1^ and 802 cm^−1^ appeared, which could be assigned to Cr-O bond [29,59]. The band of O-H and N-H stretching vibration at 3275 cm^−1^ shifted to higher wavenumbers. The intensity of N-H bending vibration at 1656 cm^−1^ slightly increased, while the bands at 1562 cm^−1^ and 750 cm^−1^ disappeared, which can be attributed to the formation of N-Cr bond [60].

In the full-range scan of the XPS spectra (Figure 10a), the common peaks of C 1s, O 1s, N 1s, Si 2p, and Fe 2p were observed before sorption. After sorption, the new peak of Cr 2p (~580 eV) appeared, which confirmed the successful sorption of Cr ions on Fe_3_O_4_@APTMS/PGME-deta. The HRES spectrum of Cr 2p with curve-fitting is shown in Figure 10b. The peaks centered at 576.5 eV and 585.8 eV could be assigned to Cr 2p_3/2_ and Cr 2p_1/2_, respectively. The Cr 2p_3/2_ peak was deconvoluted into two subpeaks at 575.8 eV and 578.1 eV that can be ascribed to the Cr(III) and Cr(VI) states. Additionally, the Cr 2p_1/2_ spectrum was fitted with two peaks located at 585 eV and 587.2 eV, which can be associated with Cr(III) and Cr(VI) species [61]. To further investigate the interactions between chromium and Fe_3_O_4_@APTMS/PGME-deta, HRES N 1s spectra of the amino-functionalized nanocomposite before and after the sorption of chromium were analyzed. The HRES N 1s spectrum before sorption (Figure 10c) was deconvoluted into two peaks at 399.2 eV and 400.2 eV, which were consistent with the presence of non-protonated primary and secondary amine groups [62]. After sorption (Figure 10d), two new peaks at 395.0 eV and 401.2 eV appeared, which can be assigned to protonated amino groups and N-Cr bond [63].

Bearing in mind that the initial solution contained only hexavalent chromium, the appearance of Cr(III) peaks in XPS spectra can be explained by the reduction of some Cr(VI) species on the Fe_3_O_4_@APTMS/PGME-deta surface during the sorption process. The chemical route behind this removal and reduction of Cr(VI) to Cr(III) by magnetic polymer nanocomposite Fe_3_O_4_@APTMS/PGME-deta was not fully explored. It is assumed that the removal mechanism for hexavalent chromium consists mostly of electrostatic attraction by the sorbents surface, electron migration, and coordination reaction [64]. Under experimental conditions, at the initial stage, chromium oxyanion could be absorbed by electrostatic attraction with hydroxyl and amino functional groups of the Fe_3_O_4_@APTMS/PGME-deta. After sorption, the surface charge of the sorbent remained positive but became less positive upon contact with the negatively charged Cr(VI) species [65]. The Cr(VI) signals found by XPS (Figure 10) result from HCrO_4_^−^ oxyanion interaction with the hydroxyl and amino groups. This interaction does not affect the oxidation state of Cr species. However, chromium can be reduced to the trivalent form in the vicinity of an electron-donating functional group, such as a hydroxyl group. Following chromium reduction, hydroxyl groups are oxidized to the carbonyl in this process. On the surface of the sorbent, Cr(III) species can coordinate with amino groups by forming covalent bonds with N atoms [66]. XPS analysis results confirmed that some Cr(VI) species become free trivalent chromium ions.

### 3.4. Quantum Chemical Calculations

Our previous works demonstrated that calculating the coordination bond energy for the chemisorption of metal ions on the polymer gives excellent agreement with the experimentally determined maximum sorption capacities [27,67]. Quantum chemical modeling of physisorption and calculation of the energy of non-covalent binding proved to help explain the binding mechanism of oxyanions to the polymer and the affinity of individual oxyanions for the polymer [21].

The nature of chromium oxyanion binding at the active sites of sorbent was studied by quantum chemical modeling. Analyzing the sorbent’s chemical composition within its unit structure shows possible binding active sites: a diethylenetriamine-derived fragment (called a “detaOH residue”), an (aminopropyl)triethoxysilane-derived fragment (called an “APTMSOH residue”) (Figure S2), as well as (aminopropyl)silane groups derived from APTMS, which coated the surface of magnetic nanoparticles before it is incorporated into the polymer in situ [68].

The introduction of the hydroxyethyl group to the deta and APTMS skeleton is very important from the point of view of supramolecular chemistry because the number of donor groups for hydrogen bonding is increased. In addition, the OH group is a better donor for hydrogen bonding than the NH_2_ group; hence, detaOH and APTMSOH species will be used to model sorption.

**Figure 11:**
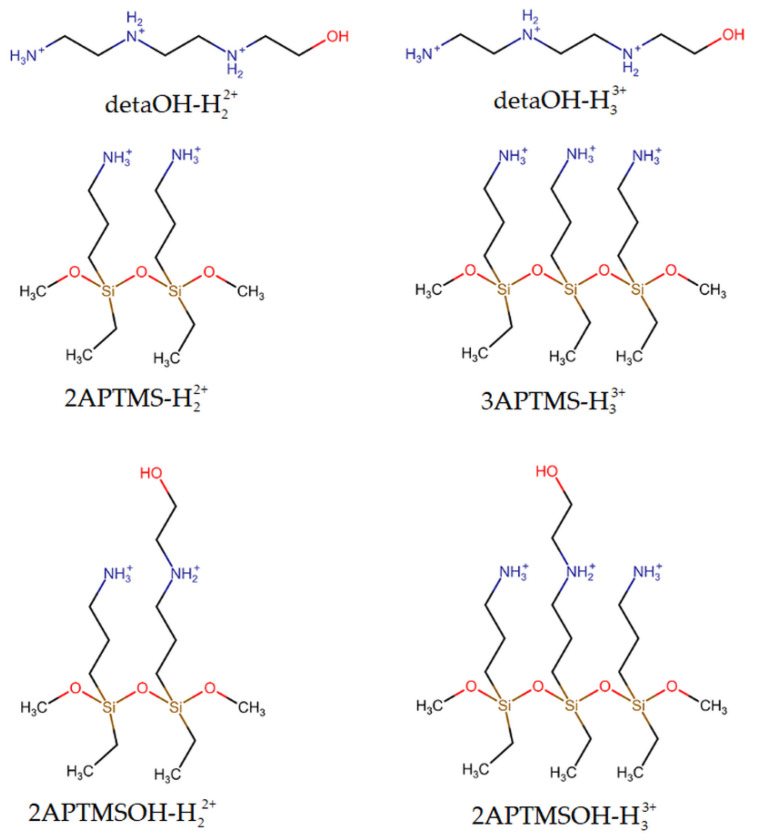
The structures of sorption sites used in model systems for estimation of the binding energies of oxyanions to polymeric nanocomposite.

Depending on the pH value, deta could be protonated (pKa values for deta are 4.42, 9.21, and 10.02), and the distribution of different ionic/neutral forms of deta (deta-H_3_^3+^, deta-H_2_^2+^, deta-H^+^, and deta) is shown in Figure S3 [69]. We assumed that the distribution of detaOH species would be similar to that of deta species because adding a hydroxyethyl group to deta should not cause drastic changes in the acid-base characteristics of detaOH. The free amino groups of (aminopropyl)silane fragments are protonated to pH less than 10 (pK_a_ = 10.6 for APTMS); hence, the positively charged surface of the silane layer is a potentially good absorbent for negatively charged oxyanions [70]. Model systems for estimation of the binding energies of oxyanions to the copolymer include all molecular fragments, which under experimental conditions, could be found on the surface of the polymeric nanocomposite (Figure 11): double and triple protonated detaOH species (detaOH-H_2_^2+^ and detaOH-H_3_^3+^), protonated species formed by condensation of two APTMS (2APTMS-H_2_^2+^) or three APTMS (3APTMS-H_3_^3+^), and their hydroxyethyl derivatives (2APTMSOH-H_2_^2+^ and 3APTMSOH-H_3_^3+^).

**Figure 12:**
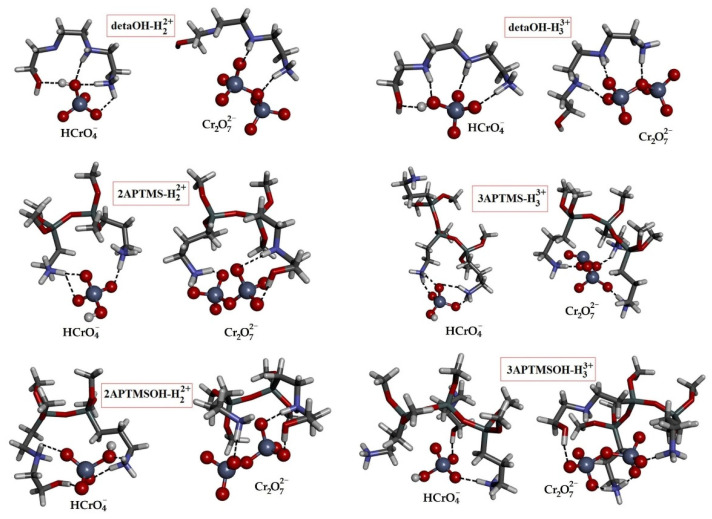
The optimized structures of dimers, used to estimate the energies of interactions between hydrogen chromate or dichromate ions and cationic sorption sites.

At pH = 5.9, the dominant species are hydrogen chromate (HCrO_4_^−^) and chromate (CrO_4_^2−^) ions and, to a lesser extent, dichromate (Cr_2_O_7_^2−^) ions. Optimized structures of combinatorial configurations of HCrO_4_^−^, CrO_4_^2−^, and Cr_2_O_7_^2−^ and six sorption sites were used as the model systems to calculate the interaction energies within the complex structure (Figure 12). The results of the calculations are shown in Table 6.

The high interaction energies resulted from strong electrostatic interactions between negatively charged oxyanion and positively charged sorption sites. In addition to electrostatic interactions, the hydrogen bonding of O-H/O, N-H/O, and C-H/O types was the cause of additional stabilization within dimer systems. The greatest contribution was attributed to N-H/O hydrogen bonds, occurring in between the positively charged amino groups and oxygen atoms from oxyanions. Sorption of oxyanion Cr_2_O_7_^2−^ species dominates over the HCrO_4_^−^ species due to higher ionic charge. However, the concentrations of Cr_2_O_7_^2−^ ions in the solution were very small, and consequently, their contribution to the overall sorption was also small.

All sorption sites (detaOH-H_2_^2+^, detaOH-H_3_^3+^, 2APTMS-H_2_^2+^, 3APTMS-H_3_^3+^, 2APTMSOH-H_2_^2+^, or 3APTMSOH-H_3_^3+^) showed stronger affinities (binding energies) to Cr_2_O_7_^2−^ ion than to HCrO_4_^−^ ion, due to the higher negative charge of dichromate ion. For the same reasons, triple-charged sorption sites (detaOH-H_3_^3+^, 3APTMS-H_3_^3+^, and 3APTMSOH-H_3_^3+^) formed stronger interactions with oxyanions compared to double-charged sorption sites (detaOH-H_2_^2+^, 2APTMS-H_2_^2+^, and 2APTMSOH-H_2_^2+^).

By comparing the energy of interactions of Cr_2_O_7_^2−^ ion with triple-charged detaOH-H_3_^3+^ sorption site (energy of −479.61 kcal/mol) and triple-charged 3APTMS-H_3_^3+^ and 3APTMSOH-H_3_^3+^ sorption sites (energies of −458.48 and −435.50 kcal/mol, respectively), it is possible to conclude at first glance that the binding of the dichromate ion to the detaOH sorption site was slightly preferred. However, we should not ignore that magnetic nanoparticles have more than three aminopropyl silane groups, indicating a greater possibility of dichromate ion binding on the surface of magnetic nanoparticles.

Quantum chemical calculations showed that the CrO_4_^2−^ ion, unlike HCrO_4_^−^ and Cr_2_O_7_^2−^ ions, undergoes neutralization after binding to the sorption sites, except to 3APTMS-H_3_^3+^ and 3APTMSOH-H_3_^3+^ sorption sites (Table S3). Namely, the double negative charge is distributed over four oxygen atoms in the case of the chromate ion, while in the case of the dichromate ion, over seven oxygen atoms. Therefore, the oxygen atoms of the chromate ion were partially more negative and, therefore, more strongly attract the amine hydrogen atoms from the sorption sites. H_2_CrO_4_ and HCrO_4_^−^ ion were formed as products of neutralization, with a lower negative charge than the initial CrO_4_^2−^ ion. As a result of neutralization, there was also a decrease in the positive charge of the sorption sites, as well as a decrease in attractive electrostatic forces. Therefore, the binding energies of the newly formed Cr(VI) species were significantly lower than the binding energies of Cr_2_O_7_^2−^ ion. However, the binding of CrO_4_^2−^ ion, which was not accompanied by neutralization (energy of −483.40 kcal/mol for 3APTMS-H_3_^3+^ and −524.79 kcal/mol for 3APTMSOH-H_3_^3+^), was significantly stronger than the binding of Cr_2_O_7_^2−^ ion (energy of −458.48 kcal/mol for 3APTMS-H_3_^3+^ and −435.50 kcal/mol for 3APTMSOH-H_3_^3+^). Accordingly, one can assume that CrO_4_^2−^ ion has a significantly higher affinity for all sorption sites than HCrO_4_^−^ and Cr_2_O_7_^2−^ ions. However, after neutralization, its binding energies decreased due to the reduction in electrostatic interactions between the newly formed species.

The mechanism of oxidation by chromium (VI) species was well known, and chromic acid, one of the most commonly used oxidizing agents, reacts with diverse substrates, among them alcohols. The selective oxidation of primary and secondary alcohols into their corresponding aldehydes (or carboxylic acids) ketones can also be achieved using polymer-supported chromic acid [71]. The used polymer has positively charged amine groups, which facilitate oxidation by forming esters with chromic acid. Calculations showed that oxyanions have the ability to bind to positively charged amine groups and ethylhydroxyl groups of the detaOH-H_2_^2+^, detaOH-H_3_^3+^, 2APTMSOH-H_2_^2+^, or 3APTMSOH-H_3_^3+^ sorbent. Accordingly, it should be expected that oxidation of the alcohol group will occur, as well as the formation of Cr(III) species. Cr(III) oxyanion, which is less toxic than Cr(VI), binds to N atoms directly by forming covalent bonds [66]. The sorption of Cu(II), Co(II), Cd(II), and Ni(II) ions by amino-functionalized chelating macroporous copolymers poly(GMA-*co*-EGDMA)-amine and sorption selectivity of the subject copolymers were successfully quantified experimentally and modeled by quantum chemical calculations [27,31,67]. According to these results, coordination of the diethylenetriamine group from Fe_3_O_4_@APTMS/PGME-deta to the Cr(III) ion can be expected.

## 4. Conclusions

This investigation showed that macroporous magnetic nanocomposite synthesized by suspension copolymerization in the presence of silanized magnetite nanoparticles and functionalized with diethylenetriamine (Fe_3_O_4_@APTMS/PGME-deta) was found to be an effective sorbent for hexavalent chromium in aqueous solutions at low concentration. Detailed characterization confirmed the presence of silanized magnetite and amino-functionalization. The obtained magnetic polymer possessed exceptional performances (excellent chemical and thermal properties), a prerequisite for successful commercialization strategies. On the other hand, reliable modeling of sorption kinetics is crucial from both scientifical and technological standpoints. The sorption half-time was approximately 2 min, with a saturation time of 40 min. Kinetic analysis indicated that the sorption rate of Cr(VI) was controlled by liquid film diffusivity and intraparticle diffusion, while equilibrium and thermodynamic studies revealed that the process is spontaneous and endothermic and that chemisorption is involved. The mechanism of sorption of Cr(VI) oxyanions was discussed through a synergy of experimental analysis and modeling approach, which accounted for the possible protonated states of the sorbent, the equilibrium forms of Cr(VI) species in solution, as well as possible non-covalent interactions responsible for their attraction. Namely, the quantum chemical calculations identified two potential binding sites for Cr(VI) oxyanions on the surface of the sorbent (deta and APTMS). The appearance of Cr(III) peaks in XPS spectra of Fe_3_O_4_@APTMS/PGME-deta after chromium sorption indicated that the Cr(VI) removal was a combination of chemical sorption (the first step in the sorption mechanism) and the conversion of Cr(VI) to Cr(III) on the sorbent surface (the second step in the sorption mechanism). Both binding sites have the ability not only to bind Cr(VI) species to the sorbent but also the possibility to reduce Cr(VI) to a less harmful form of Cr(III) ions in their reaction with the OH group from the active sites. This fact makes Fe_3_O_4_@APTMS/PGME-data nanocomposite more advanced and convenient over other sorbents, which cannot reduce Cr(VI). The wide range of polymer action, stability, and sorption diversity makes it suitable for commercial and industrial water purification. The possibility of surface modification with various functionalized groups of methacrylate-based polymers ensures multipurpose use as one of the main principles of the circular economy, a new production and consumption business model, which includes optimal use of resources, reduction in raw materials consumption, and giving second life of harmful and precious metals.

## Figures and Tables

**Table 1:** Kinetic parameters of the chromium sorption onto Fe_3_O_4_@APTMS/PGME-deta.

Kinetic Model	Parameter	Value
PFO	*Q_e_^calc^* (mg/g)	1.04
	*k*_1_ (1/min)	0.28
	*R* ^2^	0.937
	*χ* ^2^	0.091
PSO	*Q_e_^calc^* (mg/g)	1.12
	*k*_2_ (g/mg min)	0.41
	*R* ^2^	0.994
	*χ* ^2^	0.004
Elovich	α_E_ (mg/g min)	4.31
	*β_E_* (g/mg)	6.97
	*R* ^2^	0.917
	*χ* ^2^	0.081
Avrami	*n_AV_*	0.12
	*k_AV_* (1/min)	2.33
	*Q_e_^calc^* (mg/g)	1.07
	*R* ^2^	0.947
	*χ* ^2^	0.079
Fractional power	*υ* (1/min)	0.16
	*k_FP_* (mg/g min)	0.56
	*R* ^2^	0.863
	*χ* ^2^	0.140

**Table 2:** Parameters of diffusion models for chromium sorption onto Fe_3_O_4_@APTMS/PGME-deta.

Diffusion Model	Parameter	Value
IPD	*k_id_*_,1_ (mg/g min^0.5^)	0.33
	*C_id_*_,1_	0.08
	*R*_1_^2^	0.990
	*k_id_*_,2_ (mg/g min^0.5^)	0.06
	*C_id_*_,2_	0.68
	*R*_2_^2^	0.997
	*k_id_*_,3_ (mg/g min^0.5^)	0.01
	*C_id_*_,3_	0.95
	*R*_3_^2^	0.987
Bangham	*k_B_*·10^3^ (1/g)	0.54
	*α*	0.21
	*R* ^2^	0.877
LFD	*k_LFD_* (1/min)	0.02
	*C_LFD_*	−1.10
	*R* ^2^	0.943
Boyd	*R* ^2^	0.877

**Table 3:** Parameters and error function data for isotherm models obtained by nonlinear fitting for the chromium sorption onto Fe_3_O_4_@APTMS/PGME-deta.

Isotherm Model	Parameter	Value
Langmuir	*Q_m_,_L_* (mg/g)	8.22
	*K_L_*·10^3^ (g/mg)	9.26
	*R* ^2^	0.986
	*χ* ^2^	0.625
Freundlich	*K_F_* (dm^3^/g)	0.20
	*n*	1.54
	*R* ^2^	0.995
	*χ* ^2^	0.091
Temkin	*A_T_* (dm^3^/g)	1.49
	*b_T_* (kJ/mol)	3.78
	*R* ^2^	0.800
	*χ* ^2^	4.109
Dubinin-Radushkevich	*X_DR_* (mg/g)	4.38
	*K_DR_*·10^4^ (mol^2^/kJ^2^)	1.41
	*E_DR_* (kJ/mol)	59.5
	*R* ^2^	0.918
	*χ* ^2^	-
Toth	*Q_m_,_T_* (mg/g)	63.45
	*K_T_*·10^3^	3.57
	*t*	0.31
	*R* ^2^	0.992
	*χ* ^2^	0.129
Sips	*Q_m_,_S_* (mg/g)	64.13
	*K_S_*·10^3^ (mg/dm^3^)	2.80
	*m*	0.68
	*R* ^2^	0.993
	*χ* ^2^	0.112

**Table 4:** Comparison of chromium sorption capacity of various magnetic sorbents.

Sorbent *	Operating Conditions	Sorption Capacity (mg/g)	References
Cr(VI)-IIP	pH 3, dosage: 1.7 g/dm^3^, equilibrium time: 20 min, 298 K, 300 rpm, *C_i_*: 1–400 mg/dm^3^	44.86	[46]
Fe_3_O_4_@SiO_2_@DPS	pH 2.5, dosage: 2 g/dm^3^, equilibrium time: 60 min, 298 K, *C_i_*: 3–10 mg/dm^3^	4.76	[47]
Ppy@magnetic chitosan	pH 4.5, dosage: 100 mg/dm^3^, equilibrium time: 40 min, 298 K, 200 rpm, *C_i_*: 20–200 mg/dm^3^	105	[48]
EDA-MPMs	pH 2, dosage: 1 g/dm^3^, equilibrium time: 120 min, 318 K, 150 rpm, *C_i_*: 100–1000 mg/dm^3^	253.2	[49]
Fe_3_O_4_@Cr(VI)-IIP	pH 3, dosage: 10 g/dm^3^, equilibrium time: 30 min, 298 K	2.5	[50]
PPy/Fe_3_O_4_/SiO_2_	pH 4, dosage: 0.2 g/dm^3^, equilibrium time: 480 min, 298 K, *C_i_*: 25–300 mg/dm^3^	298.22	[51]
APTES@TEOS@MNP	pH 2.5, dosage: 0.5 g/dm^3^, equilibrium time: 60 min, 293 K, 60 rpm, *C_i_*: 10–100 mg/dm^3^	35	[52]
HR-M-GO/Fe_3_O_4_	pH 7, dosage: 0.5 g/dm^3^, equilibrium time: 100 min, 298 K, *C_i_*: 10–50 mg/dm^3^	31.8	[53]
PANI@MCTS	pH 2, dosage: 20 g/dm^3^, equilibrium time: 120 min, 298 K, 200 rpm, *C_i_*: 50–500 mg/dm^3^	186.6	[54]
Fe_3_O_4_-NH_2_	pH 3, dosage: 1 g/dm^3^, equilibrium time: 90 min, 298 K, 200 rpm, *C_i_*: 5–100 mg/dm^3^	232.51	[55]
Mag/CS	pH 7, dosage: 1 g/dm^3^, equilibrium time: 45 min, 303 K, *C_i_*: 30–200 mg/dm^3^	46	[56]
MNP-MPTMS	pH 4, dosage: 1 g/dm^3^, equilibrium time: 15 min, 298 K, 300 rpm, *C_i_*: 2–80 mg/dm^3^	38.61	[57]
MWCNT/Fe_3_O_4_	pH 2, mass of sorbent: 0.035 g, equilibrium time: 45 min, 298 K, 250 rpm, *C_i_*: 10–100 mg/dm^3^	76.92	[58]
Fe_3_O_4_@APTMS/PGME-deta	pH 5.9, dosage: 10 g/dm^3^, equilibrium time: 40 min, 298 K, *C_i_*: 1–180 mg/dm^3^	64.13	This study

* Cr(VI)-IIP—Magnetic Cr(VI) ion-imprinted poly(4-vinil pyridine-2-hydroxyethyl methacrylate-ethylene glycol dimethacrylate), Fe_3_O_4_@SiO_2_@DPS—1,5 diphenylcarbazide modified magnetic nanoparticles, ppy@magnetic chitosan—Polypyrrole@magnetic chitosan nanocomposite, EDA-MPMs—Magnetic poly(glycidyl methacrylate-poly(ethylene glycol) diacrylate)-ethylenediamine microsphere, Fe_3_O_4_@Cr(VI)-IIP—Magnetic Cr(VI) ion-imprinted poly[vinylimidazole-(3-aminopropyl)triethoxysilane], PPy/Fe_3_O_4_/SiO_2_—Polypyrrole/Fe_3_O_4_/SiO_2_, APTES@TEOS@MNP—Fe_3_O_4_@tetraethyl orthosilicate@(3-aminopropyl)triethoxysilane, HR-M-GO/Fe_3_O_4_—Magnetic ferrous-doped graphene, PANI@MCTS—Polyaniline@magnetic chitosan nanomaterials, Fe_3_O_4_-NH_2_—Fe_3_O_4_ nanoparticle functionalized with 1, 6-hexanediamine, Mag/CS—Magnetite/chitosan nanocomposite, MNP-MPTMS—Poly[allylamine-(N, N-dimethylacrylamide)] grafted magnetic nanoparticles, MWCNT/Fe_3_O_4_—Magnetic carbon nanotube functionalized with a carboxylic acid.

**Table 5:** Thermodynamic parameters for chromium sorption by Fe_3_O_4_@APTMS/PGME-deta.

*T* (K)	Δ*G*^0^ (kJ/mol)	Δ*H*^0^ (kJ/mol)	Δ*S*^0^ (kJ/Kmol)	*T*Δ*S*^0^ (kJ/mol)
298	−0.82	23.11	0.08	23.93
313	−1.79			24.90
328	−3.31			26.42
343	−4.44			27.55

**Table 6:** The calculated energies of interactions (in kcal/mol) between chromium oxyanion and cationic sorption site.

Cr(VI) Oxyanion	Sorbent					
	detaOH-H_2_^2+^	detaOH-H_3_^3+^	2APTMS-H_2_^2+^	3APTMS-H_3_^3+^	2APTMSOH-H_2_^2+^	3APTMSOH-H_3_^3+^
HCrO_4_^−^	−182.57	−280.48	−181.93	−227.11	−181.78	−234.04
Cr_2_O_7_^2−^	−323.84	−479.61	−410.01	−458.48	−327.35	−435.50

## Data Availability

The data presented in this study are available on request from the corresponding author.

## Supplementary Materials

The following supporting information can be downloaded at: https://www.mdpi.com/article/10.3390/ma16062233/s1, Table S1: Kinetic models equations; Table S2: Equations for isotherm models; Table S3: The calculated energies of interactions (in kcal/mol) between CrO_4_^2−^ ion and sorption sites, including the species formed in the reaction of neutralization; Figure S1: The plots for (**a**) IPD, (**b**) Bangham, (**c**) Boyd and (**d**) LFD models for removal of chromium on Fe_3_O_4_@APTMS/PGME-deta; Figure S2: The reaction of epoxy groups with deta and APTMS and formation of appropriate detaOH and APTMSOH derivatives; Figure S3: The structures and distribution of different deta forms (detaH_3_^3+^, detaH_2_^2+^, detaH^+^, and deta) depending on pH value.
