# Supplementary material for: Speciation of Hexavalent Chromium in Aqueous Solutions Using a Magnetic Silica-Coated Amino-Modified Glycidyl Methacrylate Polymer Nanocomposite
Source: Materials (Basel). 2023 Mar 10;16(6):2233. doi: 10.3390/ma16062233 (PMC10052201; doi:10.3390/ma16062233)
Supplement: Supplementary file 1 [file materials-16-02233-s001.zip › materials-2213184-supplementary.pdf]

Supplementary Materials

Speciation of Hexavalent Chromium in Aqueous Solutions Using a Magnetic Silica-Coated Amino-Modified Glycidyl Methacrylate Polymer Nanocomposite

Ljiljana Suručić [1,*], Goran Janjić [2], Bojana Marković [2], Tamara Tadić [2], Zorica Vuković [2], Aleksandra Nastasović [2] and Antonije Onjia [3,*]

[1] Faculty of Medicine, University of Banja Luka, Save Mrkalja 14, 78000 Banja Luka, Bosnia and Herzegovina
[2] Institute of Chemistry, Technology and Metallurgy, University of Belgrade, Njegoševa 12, 11000 Belgrade, Serbia
[3] Faculty of Technology and Metallurgy, University of Belgrade, Karnegijeva 4, 11000 Belgrade, Serbia
* Correspondence: ljiljana.surucic@med.unibl.org (L.S.); onjia@tmf.bg.ac.rs (A.O.)

Table S1. Kinetic models equations.

Kinetic model	Equation	Parameter
PFO	$Q_t = Q_e\left(1-e^{-k_1 t}\right)$	k_1 – the rate constant of PFO model
PSO	$Q_t = \frac{Q_e^2 k_2 t}{1+Q_e k_2 t}$	k_2 – the rate constant of PSO model
Elovich	$Q_t = \frac{1}{\beta_E}\ln\left(\alpha_E \beta_E t+1\right)$	α_E – the initial sorption constant<br>β_E – the desorption constant
Avrami	$Q_t = Q_e\left(1-\exp\left[-k_{AV} t^{n_{AV}}\right]\right)$	k_{AV} – the Avrami kinetic constant<br>n_{AV} – the Avrami fractional kinetic order
Fractional power	$Q_t = k_{FP} t^{v}$	K_{FP} – the fractional power constant<br>v – the fractional power rate constant
IPD	$Q_t = k_{id} t^{0.5} + C_{id}$	k_{id} – the rate constant of IPD model<br>C_{id} – the intercept (reflects the boundary layer impact)
Bangham	$\log\log\left(\frac{C_i}{C_i - Q_t m}\right) = \log\frac{k_B m}{2.303V} + \alpha\log t$	k_B – the constant rate of Bangham model<br>α – the constant (indicates the adsorption intensity)
LFD	$\ln(1-F) = -k_{LFD} t + C, F = \frac{Q_t}{Q_e}$	F – fraction of solute adsorbed at any time t<br>k_{LFD} – the equilibrium fractional attainment
Boyd	$F \prec 0.85,\ Bt = \left(\sqrt{\pi} - \sqrt{1-\frac{\pi F}{3}}\right)^2$<br>$F \succ 0.85,\ Bt = -0.4977 - \ln(1-F)$	Bt – the mathematical function of F

Table S2. Equations for isotherm models.

Isotherm model	Equation	Parameter
Langmuir	$Q_e = \frac{Q_{m,L} K_L C_e}{1 + K_L C_e}$	$Q_{m,L}$ – the maximum monolayer coverage capacity K_L – the Langmuir isotherm constant (binding energy of adsorption)
Freundlich	$Q_e = K_F C_e^{1/n}$	K_F – the constant of Freundlich isotherm<br>n – the Freundlich constant related to the surface heterogenity
Temkin	$Q_e = \frac{RT}{b_T} \ln(A_T C_e)$	b_T – Temkin isotherm constant related to the heat of sorption<br>A_T – Temkin isotherm equilibrium binding constant
Dubinin-Radushkevich	$Q_e = X_{DR} e^{-K_{DR} \varepsilon^2}$<br>$\varepsilon = RT \ln\left(1 + \frac{1}{C_e}\right)$<br>$E_{DR} = \frac{1}{\sqrt{2K_{DR}}}$	X_{DR} – the Dubinin-Radushkevich maximum adsorption capacity<br>K_{DR} – the activity coefficient<br>ε – the Polanyi potential<br>E_{DR} – the adsorption energy
Toth	$Q_e = \frac{Q_{m,T} K_T C_e}{\left(1 + (K_T C_e)^t\right)^{1/t}}$	$Q_{m,T}$ – the Toth maximum adsorption capacity<br>K_T – the Toth isotherm model constant<br>t – the Toth model exponent
Sips	$Q_e = \frac{Q_{m,S} K_S C_e{}^m}{1 - K_S C_e{}^m}$	$Q_{m,S}$ – the Sips maximum adsorption capacity<br>K_S – the Sips isotherm model constant<br>m – the Sips model exponent

Table S3. The calculated energies of interactions (in kcal/mol) between CrO_4^{2-} ion and sorption sites, including the species formed in the reaction of neutralization.

	Sorption sites					
	detaOH-H_2^{2+}	detaOH-H_3^{3+}	2APTMS-H_2^{2+}	3APTMS-H_3^{3+}	2APTMSOH-H_2^{2+}	3APTMSOH-H_3^{3+}
Neutralization?	YES	YES	YES	NO	YES	NO
The final product of neutralization	H_2CrO_4	H_2CrO_4	$HCrO_4^-$	CrO_4^{2-}	$HCrO_4^-$	CrO_4^{2-}
Binding energy	−30.89	−39.00	−117.12	−483.40	−112.18	−524.79

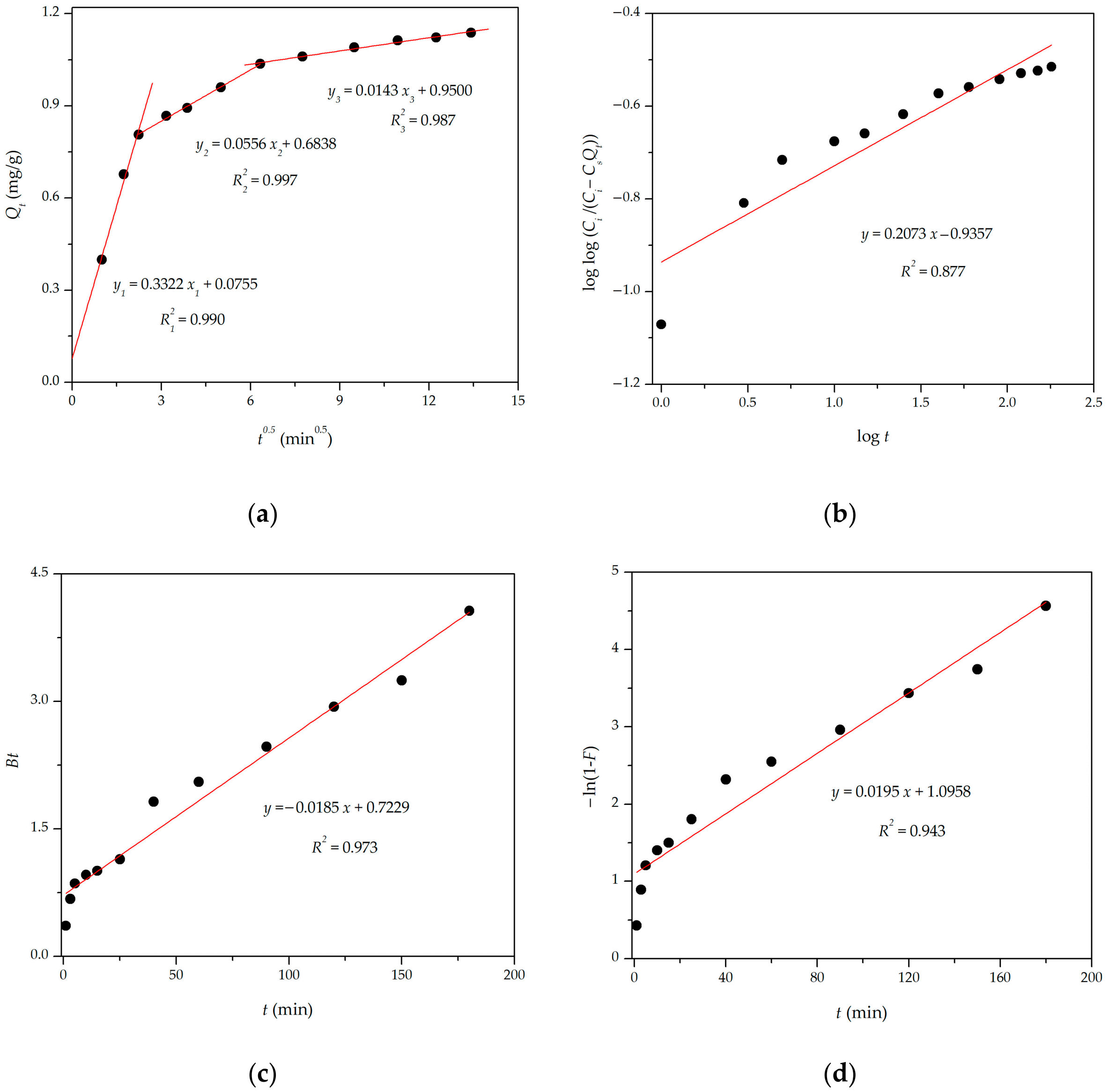

Figure S1. The plots for (**a**) IPD, (**b**) Bangham, (**c**) Boyd and (**d**) LFD models for removal of chromium on Fe_3O_4@APTMS/PGME-deta.

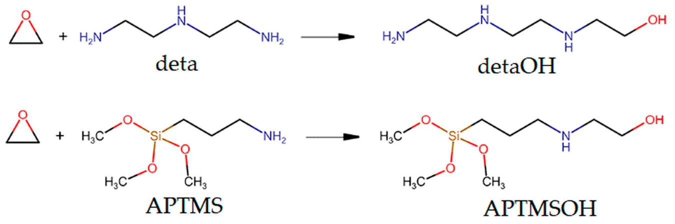

Figure S2. The reaction of epoxy groups with deta and APTMS and formation of appropriate detaOH and APTMSOH derivatives.

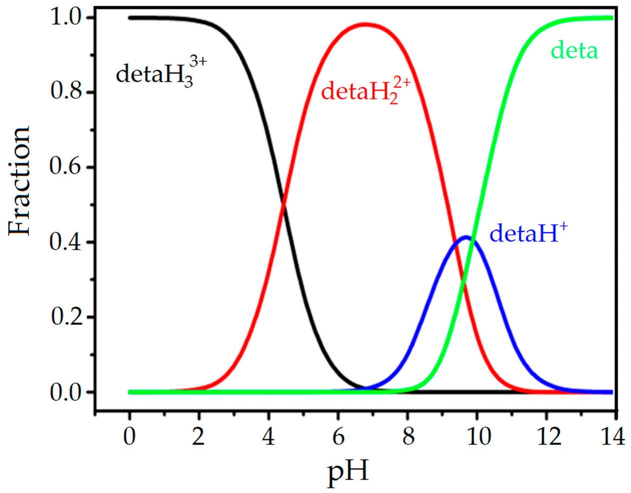

Figure S3. The structures and distribution of different deta forms ($detaH_3^{3+}$, $detaH_2^{2+}$, $detaH^+$, and deta) depending on pH value.
